# Are parenting style and Mediterranean diet in children associated?

**DOI:** 10.3389/fnut.2025.1661419

**Published:** 2025-12-15

**Authors:** Alessandra Buja, Andrea Miatton, Anna Zanovello, Filippo Brocadello, Marian Nur Muhiddin, Ilaria Spreghini, Giulia Grotto, Tatjana Baldovin

**Affiliations:** 1 Department of Cardiac, Thoracic, Vascular Sciences, and Public Health, University of Padua, Padua, Italy; 2 Affidea Poliambulatorio Morgagni, Padua, Italy; 3 Department of Pharmaceutical and Pharmacological Sciences, University of Padua, Padua, Italy

**Keywords:** Mediterranean diet, parental behavior, child habits, pediatrics, health promotion

## Abstract

**Introduction:**

The Mediterranean diet (MD) is widely recommended as a healthy eating pattern for children because of its many benefits for growth, development, and long-term well-being. Eating habits during childhood are significantly influenced by parental behavior and may lead to lasting eating habits in later life. The purpose of this study is to examine the link between three different parenting styles (social, didactic, disciplinant) and children’s adherence to the MD.

**Methods:**

A total of 332 fifth-grade students, ages 10–12, participated in a cross-sectional survey as part of an educational intervention called “Le Buone Abitudini” [Good Habits]. The children’s mothers were asked to respond anonymously to a self-administered online multiple-choice questionnaire that investigated children’s adherence to the MD (using the KidMed score) and variables related to their lifestyles, behavioral characteristics, socioeconomic factors, and Parenting Styles Questionnaire (PSQ). Multivariable stepwise linear regression was performed to test the association between children’s KidMed score and parental adherence to three types of behavior: social, didactic, and disciplinant, adjusting for covariates.

**Findings:**

According to the KidMed score, 86.2% of children in the sample had medium or high adherence to the MD. Multivariable analysis revealed a direct correlation between didactic parenting style and increased compliance with MD (regression coefficient 0.95, *p* < 0.001).

**Conclusion:**

The present study emphasizes that parental behavior can significantly influence children’s adoption of healthy habits. A didactic parenting style that combines caring guidance with the enforcement of age-appropriate rules is most effective in enhancing children’s adherence to the MD. Health promotion programs should teach parents this aspect of education to equip them with the best tools for fostering healthy eating habits in their children.

## Introduction

Recognising the importance of a complete, nutrient-rich diet is crucial for maintaining good health and well-being in childhood and later life (1, 2). Early adoption of healthy eating habits can significantly reduce the risk of developing obesity, type 2 diabetes and cardiovascular disease (3, 4). Encouraging children to eat a variety of whole foods and reducing their intake of processed foods high in sugars and unhealthy fats establishes lifelong healthy eating patterns (5, 6).

The Mediterranean diet (MD), inspired by the traditional eating habits of countries bordering the Mediterranean Sea, offers a holistic approach to nutrition that combines palatable foods with profound health benefits, making it an ideal choice for those seeking to improve their overall well-being (7, 8). This diet emphasises the consumption of whole foods, including fresh fruit and vegetables, whole grains, pulses, nuts, seeds and olive oil, while encouraging moderate intakes of fish and poultry and minimal consumption of red meat and sweets (7). Rich in healthy fats, antioxidants, vitamins and minerals, the Mediterranean diet is associated with a myriad of health benefits (9–13). In addition to its nutritional benefits, the Mediterranean diet promotes a holistic approach to food and lifestyle that enhances emotional well-being and facilitates conscious eating practices. Shared family meals and the social aspects of eating are emphasised, fostering positive attitudes towards food and mealtimes (14, 15).

It has been shown that adolescents with authoritative parents eat more fruit and vegetables at meals than those with non-authoritative parents (16, 17). Parents act as role models and guides for their children; they can also limit the availability of some foods and induce the consumption of others (18). Parenting style, i.e., the behavior and interaction style of parents with their children, influences the development of eating habits during childhood and adolescence (19, 20). The association between parenting styles and child behavior is a critical area of study, shedding light on how different approaches to parenting can significantly shape a child’s emotional, social, and cognitive development, as well as behavioral styles (21, 22).

This article explores the association between different parenting styles and child nutrition. The findings of this study may serve as a significant guide in the development of effective strategies to support health promotion programs aimed at children.

## Methods

### Participants

The present cross-sectional study derives from a survey administered in the period March–May 2023, within the framework of an educational intervention called ‘Le Buone Abitudini’ (Good Habits), underway since the 2018/2019 school year in primary schools in the province of Padua (north-eastern Italy). This program was an educational cycle designed for teachers, students, and families with the objective of promoting healthy eating habits and fostering a culture of health and well-being. Training was offered to enhance teachers’ awareness of the importance of primary prevention and health promotion, introduce the educational materials, and encourage reflection on practical classroom activities to be developed with students. The learning modules addressed different aspects of nutrition and healthy eating: the Herbs and Spices module for first- and second-grade students introduced a sensory exploration of herbs and spices; the Fruit and Vegetables module emphasized the importance of seasonal produce, fiber, and micronutrients; the Whole Grains module for third-grade students focused on understanding the food pyramid and the role of whole grains as essential energy sources; the Nuts and Seeds module for fourth-grade students highlighted foods rich in healthy fats and introduces basic knowledge of digestion; and the Legumes module for fifth-grade students explores plant-based proteins and the concept of the balanced “One Plate.” Throughout the school year, teachers were supported by a dedicated tutoring service that provided continuous professional assistance. Of the 69 state primary schools in the province, 38 were invited to participate; 14 accepted, with at least one class participating. The sampling of schools followed a convenience approach. All parents of students attending the classes involved in the evaluation study were asked to give their written informed consent for their children’s participation. In addition, the children’s mothers were asked to complete an *ad hoc*, self-administered online questionnaire anonymously. Three hundred thirty-two questionnaires were completed and returned to the study authors.

### Materials

The questionnaire contained 34 multiple-choice questions relating to both children and their parents and covered a number of factors presumed to be potentially associated with the risk of poor adherence to the MD, i.e., the social and demographic sphere, family environment, lifestyles (in addition to diet), and behavioral traits.

The lifestyle factors examined in this study included the variable ‘hours of sleep’, which encompassed both nighttime sleep and daytime naps, the amount of time spent on homework (none, less than 1 h, or 1 h or more), and whether or not the children participated in sports or other extracurricular activities.

The variable ‘adherence to the MD’ was derived from the Italian version of the KidMed Test (23). A previous study has shown that the KidMed questionnaire is a reliable tool for assessing adherence to the Mediterranean diet (24). The KidMed score ranges from −4 to 12 points based on a 16-question test. The questions were assigned a value of −1 if they had a negative connotation towards MD adherence and +1 if they had a positive connotation. Children with scores of 3 or less were classified as having low adherence to the MD, while those with scores of 4 or more were classified as having moderate to good adherence.

The second part of the questionnaire focused on socio-economic aspects related to the parents and family environment, including: the citizenship of the parent (Italian or other); the parent’s education level (middle school or lower, high school diploma, university degree); the parent’s marital status (married/cohabiting or single/separated/divorced/widowed); the parent’s health literacy was assessed by one item “The need for assistance in understanding information provided by the doctor or pharmacist (never/rarely or occasionally/frequently/always)”; and the family’s disposable income. The latter was assessed with the question, ‘How do you manage to make ends meet with your finances?’ (very easily, fairly easily, or with some/great difficulty).

The Parental Style Questionnaire (PSQ) (25, 26) was used to assess parental behavior across three core areas: social, educational, and disciplinary. The Italian version of the PSQ has been previously translated and psychometrically validated, demonstrating good reliability and construct validity in Italian populations. These areas include five, eight, and four items, respectively. Each item was rated on a 5-point Likert scale (1 = ‘never’ to 5 = ‘always’). A final score for each area was calculated by averaging the scores of the relevant items. Higher scores indicate greater levels of behavior in that area. The scores for each area were stratified into tertiles, with the first tertile distinguished from the second and third, particularly given the absence of universally established clinical cut-off points for the PSQ.

### Statistical analyses

Categorical variables were summarized using absolute frequency and percentage. For representing variables based on score violin plots were used. The difference in frequency distribution across Mediterranean adherence groups was analyzed using the chi-square test or Fisher’s exact test, with the latter only applied when the contingency tables had fewer than five total counts. Finally, a multivariable backward linear regression of the KidMed score was conducted, with backward selection of independent variables, including all lifestyle factors, socioeconomic factors related to parents’ health literacy and family environment, and parenting style domains. Missing values were handled differently depending on the variable. Whenever possible, the KidMed category (poor, medium-high) was inferred when a single answer from the KidMed questionnaire was missing. When more than one value was missing, the subject was excluded from the analysis. The same approach was applied for parenting style. For missing values in other variables, complete-case analysis was used. Results were considered statistically significant when *p* < 0.05. All statistical analyses were performed using R software (version 14).

### Ethics approval and consent to participate

This study was approved by the Ethical Committee at Padova Teaching Hospital. The children’s participation in the study was subject to the consent of the directors at the schools involved. Parents of all the children ultimately participating in the study then signed an informed consent form. All procedures complied with the ethical standards adopted by Padova Teaching Hospital, the Italian National Research Committee, and the 1964 Helsinki Declaration and subsequent revisions thereof, or comparable ethical standards. All the methods/procedures were performed in accordance with the relevant guidelines and regulations.

## Results

The final sample comprised 332 children, aged between 10 and 11 years (mean 10.25, SD 0.45), almost equally distributed between males and females (161 and 166 respectively, with 5 missing values).

A description of the characteristics of the sample is given in Table 1. Two thirds (66.6%) of the children included in the sample had a regular weight, while 4.3% were underweight and almost 30% were overweight (18.4%) or obese (10.7%). For 13.8% of the sample, adherence to MD was poor.

**Table 1:** Descriptive statistics of the sample.

Variables	Frequency (%) *N* = 332
Sex^a^	
Male	161 (50.8%)
Female	166 (49.2%)
Siblings^b^	
None	70 (21.2%)
One or more	261 (78.9%)
Time dedicated to homework	
No homework	9 (2.7%)
<1 h	99 (29.8%)
≥1 h	224 (67.5%)
Out-of-school activities^c^	
None	117 (35.6%)
One or more	212 (64.4%)
Sports^d^	
None	55 (16.6%)
One or more	276(83.4%)
Hours of sleep^e^	
Median (Q1–Q3)	9 (8–9)
Parent’s citizenship^f^	
Italian	255 (79.9%)
Other	64 (20.1%)
Parent’s marital status^g^	
Married/cohabiting	282 (89.5%)
Unmarried/divorced/widow	33 (10.5%)
Parent’s education^h^	
Middle school or less	45 (14.1%)
High school diploma	137 (42.9%)
University degree	137 (42.9%)
Family’s disposable income^i^	
Low	115 (35.8%)
Medium	146 (45.5%)
High	60 (18.7%)
Parent’s need for help in medical issues^j^	
Never/rarely	223 (70.8%)
Occasionally/frequently/always	92 (29.2%)
Family members^k^	
≤3	88 (27.2%)
4	77 (23.8%)
>4	159 (49.1%)
Adherence to the MD^l^	
Poor	45 (13.8%)
Medium/high	281 (86.2%)
BMI^m^	
Underweight	13 (4.3%)
Regular weight	199 (66.6%)
Overweight	55 (18.4%)
Obese	32 (10.7%)

Data not-available in ^a^5 (1.5%), ^b^1 (0.3%), ^c^3 (0.9%), ^d^1 (0.3%), ^e^7 (2.1%), ^f^13 (3.9%), ^g^17 (5.1%), ^h^13 (3.9%), ^i^11 (3.3%), ^j^17 (5.1%), ^k^8 (2.4%), ^l^6 (1.8%), ^m^33 (9.9%).

**Figure 1:**
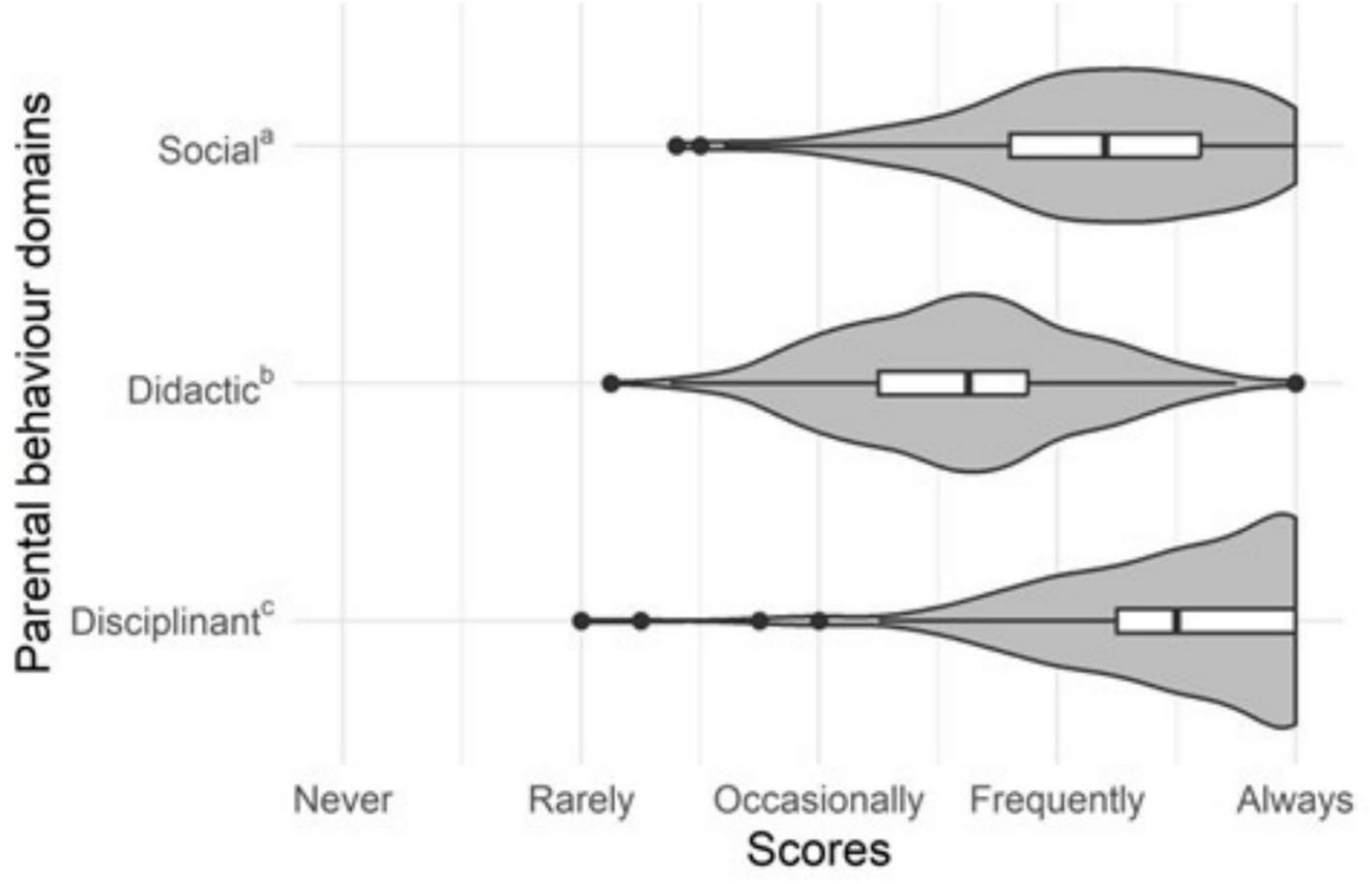
The sample’s distribution of the parental behavior domains’ scores.

Figure 1 shows the distribution of the sample according to the parenting styles questionnaire. Parents favor the adoption of disciplining behavior towards their children, while the social approach, although frequent, is practiced less and the didactic approach is reserved for a smaller number of cases.

Bivariate distributions between KidMed groups and covariates are shown in Table 2. However, multivariable regression (Table 3) showed a statistically significant association between parental teaching behavior and increased children’s adherence to the Mediterranean diet (regression coefficient 0.88, SE 0.26, *p* < 0.001).

**Table 2:** Distributions of the parental behavior domains’, the scores for media usage and time spent on online and digital activities by KidMed groups.

Parental behavior domains		Adherence to the MD		*p*-value
		Poor	Medium-high	
Parental behavior: social	First tertile	19 (15.2%)	106 (84.8%)	0.862
	Second and third tertiles	26 (13.8%)	162 (86.2%)	
Parental behavior: didactic	First tertile	18 (16.2%)	93 (83.8%)	0.616
	Second and third tertiles	27 (13.4%)	174 (86.6%)	
Parental behavior: disciplinant	First tertile	16 (14.5%)	94 (85.5%)	1
	Second and third tertiles	29 (14.2%)	175 (85.8%)	

**Table 3:** Linear stepwise backwards regression of KidMed score and parental behavior (other confounding variables entered: sex, siblings, time dedicated to homework, out-of-school activities, sports, hours of sleep, parent’s citizenship, parent’s marital status, parent’s education, parent’s health literacy, family’s disposable income, parent’s need for help in medical issues, family members).

Variables	Regression coefficient	SE	*p* value
Parental behavior: didactic (score)	0.95	0.25	<0.001
Siblings (yes vs. no)	1.38	0.52	0.009
Hours of sleep	0.59	0.16	<0.001
Family members (4 vs. ≤ 3)	−0.96	0.50	0.054
Family members (>4 vs. ≤ 3)	−1.31	0.53	0.015

## Discussion

This study examined the association between parenting style and adherence to the Mediterranean diet among students attending five grade schools. The results indicated that, among the parental behaviors assessed, didactic style was associated with greater adherence to the Mediterranean diet in children.

‘Didactic’ translates into parents who are present and attentive to their children’s education, but also supportive and involved in their children’s needs. The Parenting Style Questionnaire investigates this domain with questions such as ‘I provide my child with independent time to explore and learn on his or her own’ and ‘I provide my child with a structured, organised and predictable environment’ (26). This type of behavior is often referred to in the literature as ‘authoritative’, according to Baumrind’s 1971 definition (21, 22, 27, 28), which describes it as structured with age-appropriate demands and supervision and sensitive to the child’s feelings and wishes. Accordingly, previous studies have shown that the ‘authoritative’ parenting style is associated with long-term benefits in children’s health behaviors, compared to the ‘authoritarian’ (high expectations, less acceptance) and ‘permissive’ (freer, more forgiving) types. These benefits include a lower likelihood of experimenting with cigarettes, alcohol, or illicit drugs (29), a lower risk of being overweight (30), and higher intakes of fruit and vegetables (16, 17).

A previous Lebanese study (19) analysed the relationship between parental style and children’s adherence to the Mediterranean diet in 341 adolescents. Participants were aged between 16 and 18 years and parenting styles were assessed using their responses to the Authoritative Parenting Index (in four categories: authoritative, neglectful, permissive and authoritarian). The results showed that adolescents with neglectful parents were significantly less likely to adhere to the Mediterranean diet than those with authoritative parents, leading the authors to conclude that a firm but warm parenting style significantly increased adolescents’ adherence to the Mediterranean diet.

The present study extends these findings by highlighting the importance of a ‘didactic’ or ‘authoritative’ parental style in improving adherence to the Mediterranean diet, even among younger children aged 10–11 years attending primary school. At this age, eating behavior is typically still regulated by parental supervision and control (31), although this influence gradually diminishes as children become more independent and autonomous with age (32).

The relation between didactic or authoritative parenting styles and children’s dietary habits remains partially unclear and is currently under ongoing investigation. Nevertheless, existing literature provides valuable insights into potential mechanisms underlying this association. For example, a previous cross-sectional study explored the link between parenting styles, food-related parenting practices, and the dietary patterns of ten-year-old children (33). The findings suggested that the authoritative parenting style is positively associated with mealtime structural practices, such as the practice of sharing family meals. This finding indicates that children of authoritative parents tend to adopt healthier dietary habits by integrating specific elements of mealtime structure that are absent in other parenting styles. For instance, authoritarian parents may enforce particular mealtime practices (e.g., ensuring a child eats the same meal as others) but may not establish a supportive mealtime environment that encourages family communication, which could ultimately enhance children’s dietary quality (34). Permissive parents may choose not to impose any structure or rules concerning mealtimes, thereby allowing children to make independent decisions regarding where and when to eat. This approach may potentially lead to undesirable dietary habits (30).

Similarly, a prior review examining parents’ dietary behaviors and their potential influence on children’s eating habits concluded that family meals played a significant role in modelling children’s dietary habits, as they represent a crucial moment of control and interaction between parents and their children (35). The parental practices exerting the most influence were role modelling and moderate restrictions, indicating that increased parental encouragement and reduced excessive pressure, as exemplified by authoritative parents, could positively affect children’s dietary behaviors.

Therefore, health promotion interventions aimed at dietary changes for children should consider the role of parental behavior to be truly effective, involving also parents and families (19). Parents who recognize the importance of establishing healthy eating habits in early childhood will be better equipped to teach their children good dietary practices.

These insights can guide the creation of health promotion programs for families aimed at increasing adherence to the Mediterranean diet. Specifically, it might be beneficial to incorporate training on authoritative parenting techniques into nutrition initiatives. For instance, offering parents strategies to establish clear mealtime rules and explain the reasons behind them can foster children’s autonomous motivation, encourage family meals, and reinforce healthy eating habits.

### Limitations

This study has several limitations that need to be acknowledged. First, the analyses do not establish causality, as the cross-sectional model only allows us to discuss associations. Additionally, although the dependent variable (parental behavior) has been validated, its nature makes it challenging to address all confounding factors and provide conclusive, unbiased results.

Second, self-reported data obtained through self-administered questionnaires are subject to various biases, including sampling bias, non-response bias, and acquiescence bias. Furthermore, the topics covered in the study could be a sensitive issue for mothers, causing them to give exaggerated or minimized responses due to the influence of social desirability.

However, to our knowledge, this study is the first to investigate the association between parental behavior and the adhesion of school children to the Mediterranean diet. Given the limitations in obtaining more objective evidence on the factors influencing children’s eating behavior, we encourage future research to help fill the remaining gaps.

## Conclusion

The findings of this study underline the significance of parental behavior in shaping children’s eating habits and adherence to a healthy diet. The positive association between the didactic parenting style and children’s adherence to the Mediterranean diet could suggest that parents who balance caring guidance with appropriate rule-setting can effectively influence their children’s dietary patterns. Given the importance of early dietary habits in long-term health outcomes, these results emphasize the need for health promotion interventions that educate parents on the benefits of adopting a didactic parenting style.

## Data Availability

The raw data supporting the conclusions of this article will be made available by the authors, without undue reservation.
